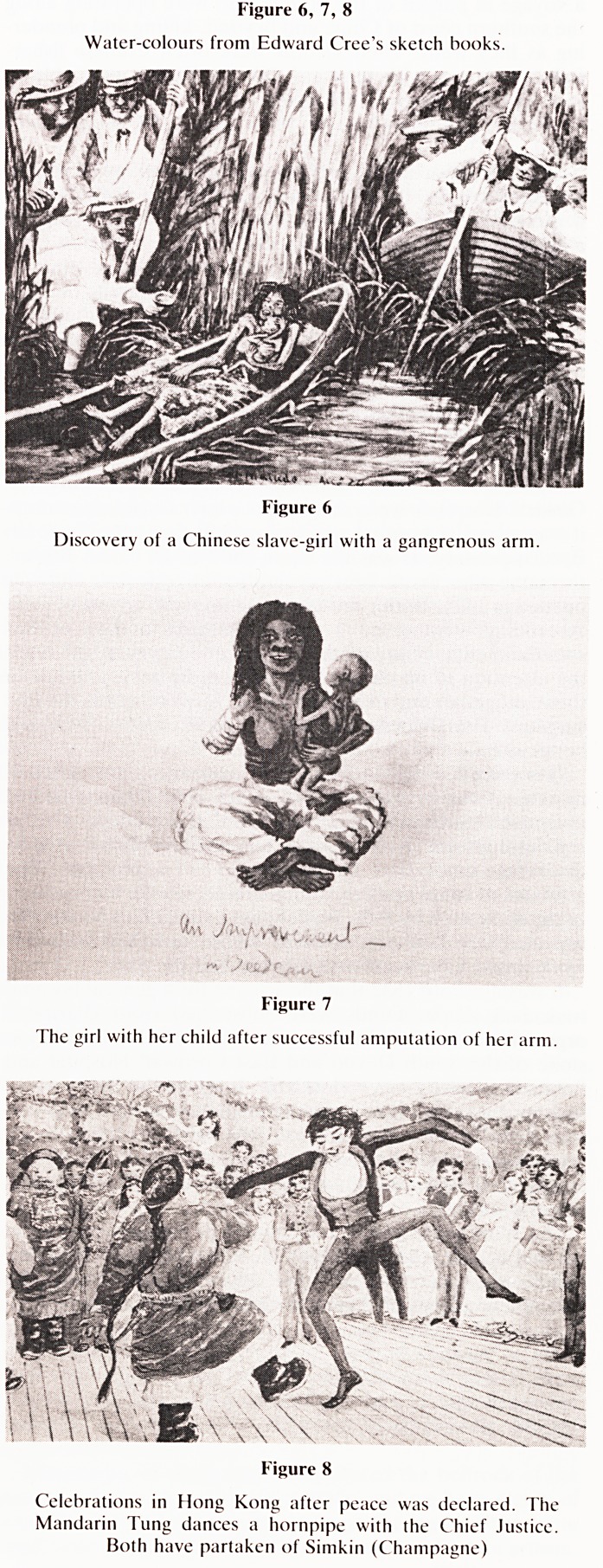# Some Plymouth Worthies (Part 2)
*Part 1 appeared in the previous number.


**Published:** 1990-06

**Authors:** Michael Reilly

**Affiliations:** Emeritus Consultant Surgeon, Plymouth


### Some Plymouth Worthies (Part 2)*

**Michael Reilly MS FRCS**
Emeritus Consultant Surgeon, Plymouth

By the end of the 18th century Plymouth and its close neighbours, Stonehouse and Devonport—'Dock', as the latter was then called—still merited the local name of 'The Three Towns'. They were separated by long sea inlets and marshy areas, now bridged or filled in. There was some rivalry between the three, but this did not extend to the medical men in practice in each town. Fifteen of them met in 1794 and formed the Plymouth Medical Society to promote professional knowledge, and later to dine together. It is either the 6th or 7th earliest Medical Society in the country, but unlike two well-known City Livery Companies, was not 'at sixes and sevens' with other Medical Societies on the score of seniority. The members were to meet at the house of one of them in rotation: this involved journeys of two or three miles through unlighted streets crowded with soldiers and sailors going to or from the wars. These were possibly the only illuminated objects around, and inclined to a little mayhem or thuggery. The members wisely chose the time of the monthly meeting for 7 p.m. on the Friday nearest the full moon. Meetings are still held on Fridays, though now more frequently. The members seem to have been of convivial bent, for in 1829 they discussed the feasibility of purchasing two stomach pumps for those in need. The proposition was lost, as it was felt that every medical practitioner should already own such an instrument.

The first president was Robert Remmett, M.D. Academically he was the best-qualified physician that Plymouth ever had, but he had a pugnacious side. This can be deduced from the portrait of him owned by the Society. He indulged in numerous political activities, and actually indicted in public three members of the Privy Council for corruption over the Treaty of Paris. Local enemies saw to it that he never became Mayor.

Most of the early members were primarily physicians, including Dr. Charles Yonge, the grandson of James. Some, however, practised surgery to a greater or lesser degree. Their interests were not confined to gastronomy, and many made contributions not only to the health of the community but in other fields.

Richard Dunning, of Plymouth Dock, was a friend and supporter of Edward Jenner, and corresponded with him frequently. In 1800 he published his 'Observations on the inoculated Cow-pox' and first used the term 'vaccination', derived from the Latin *vacca*—a cow. Jenner acknowledged his priority in the use of the word and gave him the credit at a meeting of the Royal Jennerian Society. Small-pox is now extinct, but 'vaccination' continues to be used indiscriminately for any form of immunisation. A portrait of Jenner by James Northcott, R.A., was given to Dunning by Jenner. The portrait was displayed in the Plymouth Medical Centre until it was stolen.

Stephen Hammick had a distinguished career at the Royal Naval Hospital, first as an assistant surgeon and then as a full surgeon, after an interim of four years at St. George's, London. He lectured at the Royal Naval Hospital on surgery for thirty years before leaving again for London. He was created a Baronet in 1834 and Surgeon-Extraordinary to the Royal Family. The final accolade came in 1843. In that year the Royal College of Surgeons in London was given a new Charter and became the Royal College of Surgeons of England. Under the Charter the College was to elect 300 Fellows within six months. Hammick was one of those chosen. From 1844 onwards Fellowship, apart from a few special exceptions, was only awarded after examination. Hammick subsequently became an examiner in surgery for the University of London and an original member of its Senate.

In 1824 there was an alarming epidemic in Plymouth of what was labelled 'Dockyard Disease'. It was characterised by erysipelas and lymphangitis. We would now think that it was streptococcal in origin. James Bell, a dockyard surgeon, lost his life as the result of a scratch at an autopsy on a case. Dr. John Butter wrote up the epidemic in a book entitled 'Remarks on the Irritative Fever'. All the material for the book had been collected by a Mr. Dryden, but we know nothing more about him. Butter wrote to Portsmouth to enquire about dockyard injuries there. He was told that, though injuries sometimes led to erysipelas, there had been no virulent outbreak nor death for twelve years. Sir Astley Cooper commented, in a letter quoted in the book, 'compound fractures recover in the country, but die in London'.

Cornelius Tripe, notable if only for his name, was an apprentice of Stephen Hammick. He obtained his Honorary F.R.C.S. on the recommendation of Sir Benjamin Brodie. He was also a Justice of the Peace, but nevertheless he engaged in a fist fight with James Yonge (great grandson of the famous James) over the treatment of an officer's wife. He took his place on the Bench next day with a black eye. He must, however, have been trusted by his other contemporaries. He operated on a member of a large medical family who became the great great grandmother of a gynaecologist practising until recently in the West County. He also contributed to the advancement of medicine in Plymouth by having twelve children, three girls and nine boys, three of whom became surgeons and one a dentist.

William Snow Harris was another surgical apprentice of Stephen Hammick, but turned to what is now called 'community medicine'. He was made Secretary of the Health Board in 1832, when the cholera epidemic spread to Plymouth. He was a contemporary of Michael Faraday, and his life's interest lay in electricity. In 1820 he invented a lightning conductor for use in ships, which was adopted by the Russian Navy long before the British. Unlike more recent western technological innovations acquired by Russia, this invention was acknowledged with gratitude. Czar Nicholas I presented Harris with a ring and a handsome vase of blue and gold, with a picture on it of a vessel in distress in a thunderstorm. It was inscribed 'To W. Snow Harris, Esq., from H.I.H. Nicholas Pawlowitch, Emperor of All Russia'. After this Harris' advancement was swift. He was elected F.R.S. in 1831 and awarded the Copley Gold Medal in 1835, the fourth Plymouth doctor to receive it. He was Bakerian Lecturer to the Royal Society, and was knighted in 1841 when the Royal Navy at last decided to adopt his lightning conductor. In 1860 he was made Official Scientific Referee for Her Majesty's Government on all matters electrical. He wrote many papers and books on electricity, magnetism and galvanism, but also found time, being an accomplished pianist, to take part in the musical life of Plymouth. He performed in one concert to help raise money for the town's projected new hospital.

In 1827 the resurrection-men were active, and aroused some animosity in Plymouth. This may explain why the minutes of the Medical Society in that year contain a decision not to support the defence of an Exeter surgeon accused of stealing a body from St. David's churchyard. The reason was that the grave had been so clumsily refilled that it was easily spotted.

The next year the Society sent a petition to both Houses of Parliament supporting the Anatomy Act. Interest in the study of anatomy in Plymouth seems to have persisted: a few years

* Part 1 appeared in the previous number.

ago H.M. Inspector of Anatomy gave only the second licence ever issued in this country outside a University Anatomy Department to allow dissection of cadavers at Greenbank Hospital.

There is a brief reference in the minutes of the Society to the cholera epidemic of 1832 with which Snow Harris was appointed to deal. The August meeting was deferred until October 'in consequence of the prevalence of cholera, which so occupied all the members as to render the attendance at a convivial meeting almost impossible, and certainly undesirable'.

Plymouth was grossly overcrowded and insanitary, so the infection spread fast. At its peak 476 new cases were registered in nine days, and of these 211 died. The most severe cases, and so the least likely to survive, were removed to hospital where most of them succumbed. The populace blamed the doctors for this, and relations were strained. It was believed too that corpses were used for dissection: memories of the resurrectionists were still vivid, though their activities had been curtailed.

In spite of difficulties, local doctors continued to do their best. The Rev. A.I. Coppard, who nobly helped in the epidemic, mentions in his published diary Mr. Langworthy of Plympton. He describes in detail how Langworthy, following the experience of a surgeon in Perth, gave succesful intra-venous saline infusions. The fluid was:

'Carbonate of Sodium—1 drachm
Muriate of Soda—1 drachm
Oxymuriate of Potash—6 grains

In 1 quart of water, at 110 degrees, in quantity of 4 to 7 pints'. Muriate is the former name for chloride. This solution can be calculated to be the equivalent of 0.65% saline. It is interesting to consider that this treatment was given before the importance of fluid and electrolyte balance was better understood, and to conjecture how the composition was decided. Normal saline for the human is 0.9%, but for the frog it is 0.65%—exactly what was chosen. Most students include the frog in their early biological studies, presumably following their ancestors' experimental path.

After the epidemic had died down it is pleasant to learn that the population, on appreciating the doctors' endeavours, publicly thanked them, made thirty of them Freemen of the Borough and gave to each a silver snuff-box.

One witty and delightful 19th century character, with a mind of his own, was Edward Cree. He was born in Devonport in 1814, and received private medical tuition in Bridport, Dorset, before going on to University studies in Dublin and Edinburgh. He commuted regularly, and took half his pre-qualification examinations in one and half in the other. In 1837 he obtained his Licentiate of the Society of Apothecaries. He had long decided to join the Navy rather than to go into practice in Cornwall, where he had been offered a position, but first he had to be examined as competent by the Royal College of Surgeons. As soon as he had surmounted this hurdle he was commissioned. From the moment that he joined the Navy in 1837 until his last sea-going appointment in 1861 he kept copious journals, containing some million and a quarter words, supplemented by about seventeen hundred water-colours and sketches. He never had any formal training, but took tuition from artists in Portsmouth and Malta when he was there.

One of Cree's first posts was in Stonehouse. His chief was Sir David Dickson, the senior physician. Dickson had had a distinguished naval career, but tended not to last out the day in best shape. Evening ward rounds took place at 8 p.m. Cree records that on several occasions 'old Sir David had then evidently dined, and was sometimes a little thick in his speech and very crabbed, and I have seen him feel for the pulse of the leg of an empty bed which had been put up against a wall'. While at the Royal Naval Hospital in 1837 Cree was another to treat cholera with intra-venous saline.

Cree saw service in the Mediterranean, South Africa, Ceylon, China, the Baltic and the Black sea, at Sebastopol. He recorded and sketched wherever he went (figures 6, 7 and 8). On home leave after ten years at sea he obtained his M.D. and M.R.C.S. in Edinburgh.

**Figure 6, 7, 8**

Water-colours from Edward Cree's sketch books.

**Figure 6**

Discovery of a Chinese slave-girl with a gangrenous arm.

**Figure 7**

The girl with her child after successful amputation of her arm.

**Figure 8**

Celebrations in Hong Kong after peace was declared. The Mandarin Tung dances a hornpipe with the Chief Justice. Both have partaken of Simkin (Champagne)

Cree's experiences in China are of particular interest. He was present at the first 'Opium War', which had complicated origins. Both the British and the Chinese could be held to blame, but Cree sympathised with the Chinese. His comments on his naval experiences were not always in concordance with contemporary views. He found more congenial a voyage in pursuit of the pirates who were operating along the southern coast of China and beyond, killing and plundering as they went. Their victims were often Chinese fisherfolk, and it was with their aid that the British were able to root out and destroy the fleets of two 'arch-scoundrels', Chui-apoo and Shap-'ng-tsai. More than 1,700 pirates were killed.

The story of hospitals in Plymouth, where many 'worthies' worked for the civilian population, can begin in 1703. There is a record then of a fee for £3 paid to a surgeon for the amputation of a leg in the Hospital of the Poor's Portion. This was the Workhouse, which had to find a bed from time to time for a sick inmate. The Public Dispensary was the first foundation for the sick poor in 1798. Charles Yonge was the prime mover, and later the Dispensary moved to a larger site and opened in 1809, thanks mainly to a legacy from Yonge. There was also a lying-in hospital for very poor mothers in Plymouth, and another in Devonport. These were supported by charity. The matron of the Devonport hospital was one of those who succumbed to cholera in 1832.

Apart from another Public Dispensary in Devonport, the General Hospitals were some years away. Curiously enough it was a special hospital which was built first, the Plymouth Eye Dispensary. It was the brain-child of Dr. John Butter, the same who wrote later on the 'irritative fever', and was opened in 1821. Butter must have ignored the advice of John Abernethy, who wrote 'I cannot eulogise for Eye or Ear establishments, or any other divisions of Surgery, which leads the attention to be directed *partially* to diseases, and not to them altogether and in general'. J.M. Luscombe was the first surgeon. The Dispensary moved several times to new and better sites.

It is recorded that in 1861 a new instrument, the ophthalmoscope, was in use only ten years after Helmholtz had invented it. Such expedition is unheard of nowadays. 'Creeping development' is the current bureaucratic phrase used to discourage innovation. Doctors would call it 'progress'. The attitudes of some modern administrators invoke the memory of the dedication by P.G. Wodehouse in one of his books: 'To my daughter Leonora, without whose unfailing help this work would have been completed in half the time'.

It was not until 1832 that the scheme for a general hospital took real shape. Funds were subscribed from charitable organisations and individual sources, and the foundation stone of the South Devon and East Cornwall Hospital and Public Dispensary was laid in 1835. It opened in 1840 with 12 beds and was enlarged in succeeding years to accommodate over 100. In 1877 the decision was made to move to its present site, where it is now known, for short, as Greenbank Hospital. It had cost almost £39,000—all raised by public subscription.

The Workhouse had already moved to the same area close by, and became the City Hospital. It is now called Freedom Fields, after the name of the adjacent park where the Roundhead troops of Plymouth defeated the Cavaliers.

In Devonport a later start was made, but progress was more rapid. A meeting was held in 1861 to consider and confirm the idea of a hospital. Building had already begun when the Fourth Earl of Mount Edgcumbe laid the foundation stone in 1862, and in 1863 the hospital opened under the name of the Royal Albert Hospital and the patronage of the Prince Consort.

John Wilson, M.D., M.R.C.S., worked there as a young surgeon and gained praise for his efforts during the smallpox epidemic which had then started in the Dockyard. He died in 1872, one of many surgical victims of septicaemia contracted from pricking a finger while operating. His memorial tablet was one of only two saved from destruction when, after the inception of the N.H.S., all reminders of past benefactors were being systematically eradicated. It is now in the Chapel at Derriford Hospital.

Another hospital, in Lockyer Street, began as a Homoeopathic Dispensary in 1870. It was staffed entirely by homoeopathic practitioners, with whom allopaths refused to mix. The homoeopaths soon left, it is uncharitably said, for more lucrative fields in Torquay and London. The building was then used for gynaecology.

The last important hospital to be built in Plymouth during the 19th century was at Mount Gold in 1892. It was designed for infectious diseases, and W.J. Square, one of a distinguished medical family in Plymouth, planted trees around it to protect those living in the vicinity from infection. Square, if no epidemiologist, was an accomplished surgeon. He was the first surgeon to be elected to the staff of the South Devon Hospital, and at a soirée of the Plymouth Medical Society in 1867, before Lister's work became known, presented the results of 32 operations for the stone. There had been only one fatality.

This disquisition opened with James Yonge: he should be given the last word. One of his surgical apophthegms was 'Ita quinquam derelinquatis aegros, semper sperate salutem'. This can be freely translated 'Never give up with a patient, where there's life there's hope'.

Thanks and acknowledgements for generous help are extended to:

Commander P. Yonge, R. N., for the opportunity to study his ancestor's original books and manuscripts.

Brigadier H. Cree, for making the 21 volumes of his grandfather's sketches available to be viewed.

Surgeon-Captain D. A. Lammiman, FFARCS., for providing a copy of 'The History of the Royal Naval Hospital, Plymouth' by Surg. Capt P. D. G. Pugh, OBE., FRCS and for allowing photographs to be taken at the RNH.

The late Mr. G. E. Larks, FRCS., for comprehensive notes on the history of medicine in Plymouth and on the PMS.

Dr. J. L. Stafford, for bringing the archives of the PMS to light.

Mr. E. H. Cornelius and his Library Staff at the Royal College of Surgeons.

Mr. T. M. W. McCausland, of the Department of Photography, Derriford Hospital, Plymouth.

Messrs. Webb & Bower, of Exeter, Publishers of 'The Cree Journals' edited by Michael Levien, for permission to use extracts from the book.

## Figures and Tables

**Figure 6, 7, 8 f1:**